# Salt and Brazilian ancestry

**DOI:** 10.1590/S1516-31802001000400001

**Published:** 2001-07-07

Salt and humanity are very old companions. The agricultural revolution started ten thousand years ago, and salt was responsible for where human populations settled because of its food conservation properties.

Populations like the Yanomani that do not use salt for preparing food do not like it when salt is added in, as was the case with the Yanomani in the 1980's. However, after three months of eating salted foods, people become addicted to salt and it is impossible to take the salt out from their diet. So, everyone in our modern civilization is a salt addict. This has also happened with the Yanomani, who now add much more salt in their food preparation.

Evidence for the association between salt and hypertension has been found in ancient Chinese manuscripts and throughout history. During the twentieth century, a lot of information was accumulated regarding salt and hypertension but one of the most important studies about this association was produced in the 1980's: the Intersalt Study.^1^

Intersalt was a multicentric study involving 52 centers in 32 different countries. The study included 200 people in each center, making a total of 10, 000 men and women. Four remote populations were included in this study (Yanomani, Xingu, and from Kenya and Papua-New Guinea). In these four centers salt ingestion measured by sodium excretion in 24-hour urine samples was lower when compared to other centers. People from the four remote populations also had a lower body mass index (BMI), more daily physical activity and a lower ingestion of alcohol. Intersalt concluded that a salt ingestion of 100 mmol less every day over a long time represented 2 to 3 mmHg less in blood pressure.

What is the significance of these numbers? For each individual, very little but for populations this is very important. When associated with other factors such as lower BMI, alcohol intake and increased physical activity, the decrease in systolic blood pressure can reach 9 mmHg, which represents a reduction of 16% in the prevalence of coronary heart disease, 23% in stroke and 13% in all-cause mortality.

Intersalt concluded that although there are a lot of factors influencing blood pressure levels, salt is still a very important one. Another conclusion was in relation to the Epstein-Eckoff curve, which generally shows an increased prevalence of high blood pressure levels with increasing age. Until the Intersalt data, this fact was considered normal for all populations. The four remote populations in Intersalt proved this is not correct. Blood pressure levels do not increase with age for these populations.

## Why this is so important for Brazila?

Cardiovascular mortality is the leading cause of death in Brazil.^2^ In some places like the Southeast, most of the cardiovascular deaths are from coronary heart disease. However, in the Northeast, most of the mortality is due to stroke. The most important risk factor for stroke is high blood pressure. The Brazilian heritage originates from Portugal and Portuguese tradition includes a lot of salt in foods like codfish, sausages and other very tasty dishes that form part of our lives. Bread in the Portuguese tradition also has a lot of salt, and Brazilian bakeries follow the Portuguese influence. So the Epstein-Eckoff curve for the Brazilian population in selected data is very similar to the Portuguese curve: there is a sharp increase in blood pressure levels according to age.

So, possibly, hypertension is the most important risk factor for cardiovascular disease in Brazil, with more relevance than cholesterol levels.

Brazilian people are salt-addicted like almost every other people in the world, but with a high slope on the Epstein-Eckoff curve because of our Portuguese heritage. What is the point? Salt is still an important causal factor in the genesis of hypertension, although a lot of other factors can also interfere. Industrialized foods also have a lot of salt used for conservation, as does our Portuguese heritage. Hypertension control in Brazil should include a change in our habits regarding salt intake. This is the most difficult thing: it is very hard to change habits and traditions that come from our mothers.

In a not so good translation from Otto Lara Rezende, "…an unrevealed ailment, not like those in the sonnets but as found in any disease, is always a matter for tranquillity. What you don't know, you don't have. As a diagnosis of high pressure is not followed by treatment or assistance, it is for many ordinary people just an alarm. The fat and frolicsome widow who has a dull day-by-day life is invited to cut out the salt from her rice and beans…"
